# Mechanism and Role of the Neuropeptide LGI1 Receptor ADAM23 in Regulating Biomarkers of Ferroptosis and Progression of Esophageal Cancer

**DOI:** 10.1155/2021/9227897

**Published:** 2021-12-30

**Authors:** Chen Chen, Jun Zhao, Jing-ni Liu, Chenyu Sun

**Affiliations:** ^1^Department of Thoracic Surgery, The First Affiliated Hospital of Soochow University, Medical College of Soochow University, 899 PingHai Road, Suzhou 215000, China; ^2^Department of Thoracic Surgery, Huai'an First People's Hospital, Nanjing Medical University, Huai'an, 223300 Jiangsu, China; ^3^Department of Burn and Plastic Surgery, Huaian First People's Hospital, No. 1 West Huanghe Road, Huaiyin District, Huai'an City, Jiangsu Province, China; ^4^AMITA Health Saint Joseph Hospital Chicago, 2900 N. Lake Shore Drive, Chicago, 60657 Illinois, USA

## Abstract

**Background:**

According to recent studies, ferroptosis is closely related to the efficacy and prognosis of tumour treatment. However, the role of ferroptosis in esophageal squamous cell carcinoma (ESCC) has not been explored comprehensively.

**Materials and Methods:**

The esophageal cancer (EC) transcriptome data was downloaded from The Cancer Genome Atlas (TCGA), then analyzed, to obtain the differentially expressed messenger RNA (mRNA), microRNA (miRNA), and long noncoding RNA (lncRNA) between groups with the low and high Ferroptosis Potential Index (FPI) and construct a ferroptosis-associated ceRNA network. In addition, the expression of ARHGEF26-AS1 and miR-372-3p in ESCC cell lines was assessed, and the appropriate cell lines were selected. The interaction between ARHGEF26-AS1, miR-372-3p, and ADAM23 was also determined through a dual-luciferase reporter assay. Moreover, the Western blot, Cell Counting Kit-8 (CCK-8), wound healing, cell viability, and cell death assays were conducted to establish the biological functions of the ARHGEF26-AS1/miR-372-3p/ADAM23 pathway in ESCCs.

**Results:**

An FPI scoring model reflecting the activity of the ferroptosis pathway was constructed, and a ferroptosis-associated ceRNA network was established. The findings revealed that low expression of ADAM23 and ARHGEF26-AS1 as well as high expression of miR-372-3p was associated with poor prognosis and a lower FPI score in EC patients. Functionally, overexpression of ADAM23 and ARHGEF26-AS1 and the miR-372-3p inhibitor not only promoted ferroptosis in ESCC cells *in vitro* but also inhibited the proliferation and migration of cells. Mechanistically, ARHGEF26-AS1 upregulated the expression of ADAM23 by competitively binding to miR-372-3p.

**Conclusions:**

The study showed that the lncRNA, ARHGEF26-AS1 acts as a miR-372-3p sponge that regulates the neuropeptide LGI1 receptor ADAM23 expression. This in turn not only inhibits the proliferation and migration of ESCC cells but also upregulates the ferroptosis pathway. A neuropeptide-related ferroptosis regulatory pathway was identified in this study.

## 1. Introduction

Esophageal cancer (EC) is a common malignant tumour of the gastrointestinal tract and is associated with a high mortality rate. However, despite the advancements in multidisciplinary and integrated treatment methods including surgery and radiotherapy, the overall survival (OS) of EC patients is still low [1, 2]. In addition, approximately 30-60% of patients with EC suffer from tumour recurrence within 2-3 years of systematic treatment and often have a poor prognosis [3]. Notably, ferroptosis, is a mechanism of cell death that is caused by the loss of glutathione peroxidase 4 (GPX4) activity, leading to the damage of the cell membrane [4]. According to recent studies, ferroptosis is closely related to the efficacy and prognosis of tumour treatment [5]. However, the role of ferroptosis in EC has not been explored comprehensively.

Additionally, the regulatory role of microRNA (miRNA) in tumours has been studied extensively over the past decades [6]. In oesophageal squamous cell carcinoma (ESCC), numerous microRNAs have been identified as markers of cancer [7]. Moreover, the long noncoding RNA- (lncRNA-) miRNA-messenger RNA (mRNA) regulatory pathway has been widely studied over the recent years. It was also reported that lncRNAs may act as competitive endogenous RNAs (ceRNAs) that competitively suppress the expression of miRNA, ultimately leading to the deregulation of mRNA by miRNAs [8]. However, the ceRNA network that regulates the ferroptosis pathway in ESCC is unknown.

The Ferroptosis Potential Index (FPI) is a reliable indicator of the activity of the ferroptosis pathway [9]. In addition, FPI was shown to be associated with tumour subtypes as well as clinical features and impacts sensitivity to antitumour drugs. Moreover, the single-sample Gene Set Enrichment Analysis- (ssGSEA-) based FPI scoring system was shown to reveal overall alterations in ferroptosis-regulated genes at multiple histological levels and the underlying mechanisms of ferroptosis [9]. Consequently, the present study determined the differentially expressed mRNA, miRNA. and lncRNA between groups with low and high FPI, using EC data from The Cancer Genome Atlas (TCGA), then constructed a ferroptosis-associated ceRNA network, accordingly.

This study is aimed at investigating the role of the ceRNA network in regulating the ferroptosis pathway in EC and exploring possible new therapeutic targets for the cancer. The findings revealed that ARHGEF26-AS1 acts as a miR-372-3p sponge that regulates the neuropeptide LGI1 receptor ADAM23 expression, thereby inhibiting the proliferation and migration of ESCC cells as well as upregulating the ferroptosis pathway.

## 2. Materials and Methods

### 2.1. Data Collection and Establishment of the Ferroptosis Index Model

Raw sequencing data (including miRNAs, lncRNA, and mRNA expression data) and clinical parameters from 184 cases of EC were downloaded from the TCGA database (https://portal.gdc.cancer.gov/). In addition, data on the expression of core genes related to ferroptosis was used to create an index that represented the level of ferroptosis, as previously described [9]. The ferroptosis-related genes included FDFT1, HMGCR, COQ10A, COQ10B LPCAT3, ACSL4, NCOA4, ALOX15, GPX4, SLC3A2, SLC7A11, NFE2L2, NOX1, NOX3, NOX4, and NOX5. Thereafter, the “GSVA” package in R was used to calculate the gene set enrichment scores for the ferroptosis-related genes and obtain the FPI through normalization [9, 10]. The FPI was then used to calculate the ferroptosis potential in tissue samples.

### 2.2. Identification of FPI-Associated Genes and Gene Enrichment Analysis

Patients were sorted in ascending order according to the FPI score. Thereafter, the top 50% patients were categorized into the high FPI phenotype score group while the remaining were grouped into the low FPI phenotype score group. Differential analysis was then performed using the “limma” package in R to identify the differentially expressed mRNAs, miRNAs, and lncRNAs between the low and high FPI phenotype score groups. These differentially expressed genes were defined as the FPI phenotype-associated genes (fold change > 1.0 and adjusted *p* value < 0.05). Moreover, enrichment analysis was conducted on the screened Differentially Expressed mRNAs (DE-mRNAs), including the Kyoto Encyclopedia of Genes and Genomes (KEGG) pathway analysis and Gene Ontology (GO) functional analysis, using the Database for Annotation, Visualization and Integrated Discovery (DAVID) [11].

### 2.3. CeRNA Network Prediction

The ceRNA network (consisting of DE-mRNAs, DE-miRNAs, and DE-lncRNAs) was constructed as previously described [12–15]. Interactions between DE-lncRNAs and DE-miRNAs were then confirmed using the miRcode database [16]. Moreover, potential associations between DE-mRNAs and DE-miRNAs were predicted using the miRWalk (http://mirwalk.umm.uni-heidelberg.de/), miRDB, TargetScan, and miRanda databases [17].

### 2.4. Western Blotting

In order to determine the expression levels of proteins, cells were lysed using the radioimmunoprecipitation assay (RIPA) buffer enriched with a protease inhibitor cocktail. Afterwards, total protein was quantified using the bicinchoninic acid (BCA) kit (Pierce, Rockford, IL, USA). Thereafter, 8 *μ*g of the total protein was aliquoted and sodium dodecyl sulphate-polyacrylamide gel electrophoresis (SDS-PAGE) was employed to resolve the proteins. The fractionated proteins were then electroblotted onto 0.45 *μ*m photovoltaic (PV) membranes (Immobilon™; Merck Millipore, Darmstadt, Germany). Following this, the membranes were blocked and probed overnight with the following antibodies; anti-GPX4 (#ab125066; Abcam, USA), anti-SLC3A2 (#ab108300; Abcam), anti-SLC3A2 (#ab175186; Abcam), and anti-ADAM23 (#ab126275, Abcam). *β*-Actin (#ab8226, Abcam) was used as the internal reference. Thereafter, the membranes were conjugated with horseradish peroxidase- (HRP-) conjugated secondary antibodies (#ab205719, Abcam) through incubation. Enhanced chemiluminescence was then used to detect the bound antibodies. Finally, the Image J software (NIH, Bethesda, MD, USA) was employed to quantify the intensities of the bands. The details of primer sequences can be found in the document “Supplementary methods and figures.”

### 2.5. Cell Culture

The human EC cell lines (Ec9706, TE-1, and EC109) and normal human oesophageal epithelial cells (Het-1A) were cultured in the RPMI 1640 medium with 10% fetal bovine serum (FBS) and 1% streptomycin/penicillin in a humidified incubator containing 5% CO_2_ at 37°C. All cells were purchased from LAMI Bio, Shanghai, under the brand name of The American Type Culture Collection (ATCC, Manassas, VA, USA). The medium and FBS used in this experiment were purchased from Gibco, USA.

### 2.6. Lentivirus Transfection

Logarithmic growth phase TE-1 cells were inoculated in 6-well cell culture plates at a density of 1 × 10^5^ cells per well. The medium was then changed after the cells had fused to 60%. Thereafter, the cells were transfected with the ADAM23 overexpression vector (ADAM2-OE group), LINCARHGEF26-AS1 overexpression vector (LINCARHGEF26-AS1-OE group), and an empty vector (EV group), following instructions on the Lipofectamine TM2000 kit. The details of ADAM23 can be found in the document “Supplementary methods and figures”. In addition, the cells were replaced with fresh medium after 12 h of transfection, then incubated for 48 h. Afterwards, the cells were collected for subsequent experiments.

### 2.7. Transfection of miRNAs

Briefly, chemically synthesized miRNA mimics and inhibitors (GenePharma, Shanghai, China) were used to enhance and inhibit the function of miR-372-3p. Sequences are as follows: miR-372 mimic 5′-AAAGU GCUGCGACAUUUGAGCGUGCUCAAAUGUCGCAGCACUUUUU-3′, and miR-372 inhibitor 5′-ACGCUCAAAUGUCGCAGCACUUU-3′ (both purchased from Shanghai Genepharma Company). After 24 h of inoculation, miR-372-3p was transfected for 24 h using the riboFECT™ CP transfection kit, following manufacturer's protocol (RiboBio, Guangzhou, China).

### 2.8. The Luciferase Reporter Assay

Wild type or mutant fragments of LINCARHGEF26-AS1 and ADAM23 3′UTR containing the putative-binding site for miR-372-3p were inserted into a dual-luciferase reporter vector (Promega, Madison, WI, USA). Thereafter, the TE-1 cells were placed in 96-well plates, then cotransfected with the Lipofectamine 3000 transfection reagent (Invitrogen) for miR-ctrl or miR-372-3p and the constructed luciferase reporter plasmid. Afterwards, relative luciferase activity was assayed using a dual-luciferase reporter gene assay (Promega), 48 h after transfection.

### 2.9. The Cell Counting Kit-8 (CCK-8) Assay

The cell counting kit-8 (Vazyme, China) was used to assess cell viability. Briefly, the TE-1 cells were inoculated in 96-well plates at a density of 1 × 104 cells/well. Thereafter, about 10 *μ*L of CCK-8 solution was added into each well at different time intervals (0, 24, and 48 h). The plates were then incubated for 1 h in the dark. Afterwards, absorbance at 450 nm was measured using an enzyme marker (BioTek, USA). Each set of experiments was repeated thrice.

### 2.10. The Cell Viability and Cell Death Assays

Cell viability was assessed using the Promega Cell Titer96 aqueous solution (G3580, Madison, WI, USA), as previously described [18]. On the other hand, the cell death assays were performed through Annexin V-FITC (fluorescein isothiocyanate)/7AAD (BD Pharmingen) analysis. The number of dead cells was analyzed by counting the number of cells that stained positive for Annexin V-FITC and 7-ADD.

### 2.11. Statistical Analysis

All the data is expressed as the mean ± standard deviation (SD). In addition, comparisons were made through the *t*-test or one-way analysis of variance (ANOVA). Statistical analysis was performed using the GraphPad Prism 5.01 software (GraphPad, USA). Moreover, survival analyses were conducted using Kaplan-Meier curves, and *p* values < 0.05 were considered to be statistically significant.

## 3. Results

### 3.1. The Ferroptosis Index Model and FPI-Associated Differentially Expressed Genes

**Figure 1:**
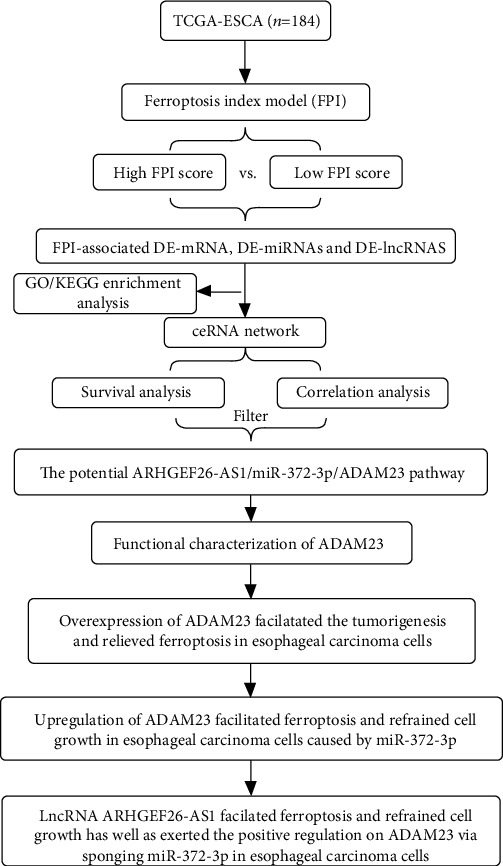
A flow chart of the study.

**Figure 2:**
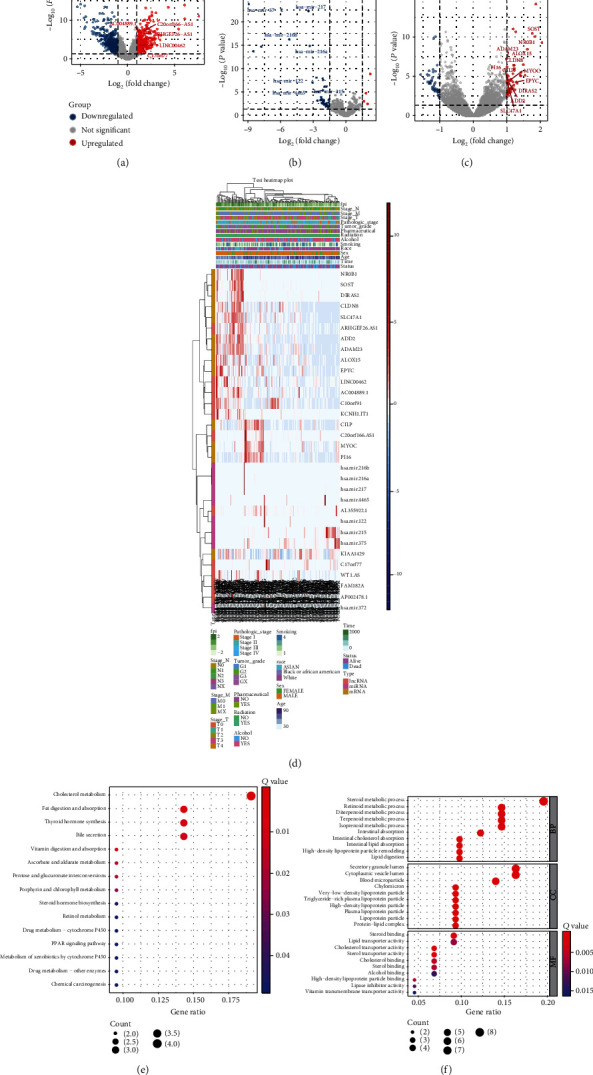
Establishment of the ferroptosis index model and identification of FPI-associated differentially expressed genes. (a–c) Volcano plots showing the (a) DE-lncRNA, (b) DE-mRNA, and (c) DE-miRNA. (d) A heatmap of the key DE-lncRNA, DE-mRNA, and DE-miRNA. (e) KEGG pathway enrichment analysis and (f) GO function analysis of the DE-mRNA.

A flow chart of the study is shown in Figure 1. The ferroptosis index model was built from the expression data of the core genes associated with ferroptosis, as previously described [9]. Additionally, patients were sorted in ascending order according to the FPI score. Thereafter, the top 50% patients were grouped into the high FPI phenotype score group, and the remaining were classified into the low FPI phenotype score category. Differential analysis was then performed to identify DE-mRNAs, DE-miRNAs, and DE-lncRNAs between the low and high FPI phenotype score groups. The volcano plot in Figures 2(a)–2(c) shows the differential expression of DE-lncRNA, DE-mRNA, and DE-miRNA. Moreover, heatmaps of the key DE-lncRNA, DE-mRNA, and DE-miRNA in combination with clinical features are shown in Figure 2(d). The results revealed that the GO functions were mainly associated with cholesterol metabolism, fat digestion and absorption, thyroid hormone synthesis, and bile secretion (Figure 2(e)). On the other hand, the KEGG pathways were mainly associated with the steroid metabolic process, retinoid metabolic process, secretory granule lumen, cytoplasm, and blood microparticle (Figure 2(f)).

### 3.2. The ceRNA Network and Clinical Correlation Analysis

**Figure 3:**
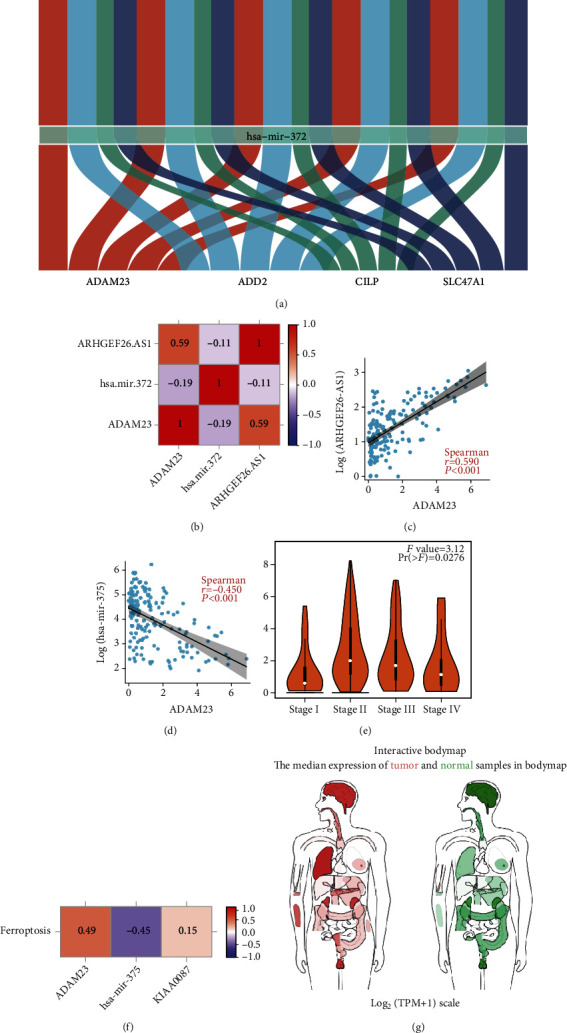
Clinical correlation analysis. (a) A ceRNA network of esophageal cancer and ferroptosis. (b) Correlations among ADAM23, miR-372, and ARHGEF26-AS1. (c) The correlation between ADAM23 and ARHGEF26-AS1 (*p* < 0.0001). (d) The correlation between ADAM23 and hsa-miR-372 (*p* < 0.001). (e) Differential expression of ADAM23 in different clinical stages. (f) Association of ADAM23, miR-372, and ARHGEF26-AS1 with FPI. (g) Distribution of ADAM23 expression in human tissues.

A ceRNA network of EC and ferroptosis (consisting of DE-mRNAs, DE-miRNAs, and DE-lncRNAs) was constructed in this study (Supplementary Figure 1A). Survival analysis revealed that low expression of MYOC, SOST, ADD2, ADAM23, C17orf77, C20orf166-AS1, and LINC00462 was associated with poor prognosis. Additionally, high expression of hsa-miR-372, hsa-miR-4465, hsa-miR-375, and hsa-miR-122 was also associated with poor prognosis (Supplementary Figure 1B). Therefore, a ceRNA network with more potential regulatory relationships was constructed (Figure 3(a)). Correlation analysis revealed that ARHGEF26-AS1 was positively associated with the expression of ADAM23 while miR-372 was negatively related to the expression of both ADAM23 and ARHGEF26-AS1 (Figures 3(b)–3(d)). Consequently, a potential ARHGEF26-AS1/miR-372-3p/ADAM23 regulatory pathway was identified. Moreover, clinical correlation analysis showed that the expression of ADAM23 was associated with clinicopathological staging (Figure 3(e)). In addition, ADAM23 and ARHGEF26-AS1 were positively correlated with the FPI score (Figure 3(f)) while miR-372-3p was negatively associated with the FPI score. Furthermore, ADAM23 and ARHGEF26-AS1 were positively correlated with ferroptosis while miR-372-3p was negatively associated with ferroptosis (Figure 3(f)). The interactive body map images in Figure 3(g) was created using GEPIA (Gene Expression Profiling Interactive Analysis) [19]. The interactive body map images of ADAM23 in human tissues also suggested that ADAM23 was enriched in the oesophagus (Figure 3(g)).

### 3.3. Functional Characterization of ADAM23

The results of GO functional analysis and GSEA revealed enrichment of ADAM23-related signaling functions, including 4 iron and 4 sulfur cluster binding, cellular response to iron ions, cellular homeostasis of potassium ions, cellular homeostasis of sodium ions, chaperone-mediated autophagy, the Major Histocompatibility Complex (MHC), and sodium ion export across the plasma membrane (Supplementary Figure 2A). Furthermore, KEGG pathway analysis revealed enrichment of IMriskScore-related pathways, including 2-oxcarboxylic acid metabolism, the AMPK signaling pathway, apoptosis, focal adhesion, the PI3K-Akt signaling pathway, the FoxO signaling pathway, the PPAR signaling pathway, the TNF signaling pathway, and differentiation of Th17 cells (Supplementary Figure 2B). The results of GO functional enrichment and KEGG pathway enrichment analyses therefore suggested a potential link between the expression of ADAM23 and biological functions such as apoptosis and ferroptosis. Moreover, correlation analysis revealed that the expression of ADAM23 was significantly associated with the ferroptosis-related genes, GPX4, SLC3A2, and SLC7A11 (Supplementary Figure 2C-E). This further indicated a potential association between ADAM23 and ferroptosis. However, survival analysis suggested that the mRNA expression of GPX4, SLC3A2, and SLC7A11 had no significant association with the prognosis of EC patients (Supplementary Figure 2F-H).

### 3.4. Overexpression of ADAM23 Inhibited Tumorigenesis and Facilitated Ferroptosis in Oesophageal Carcinoma Cells (ECC)

**Figure 4:**
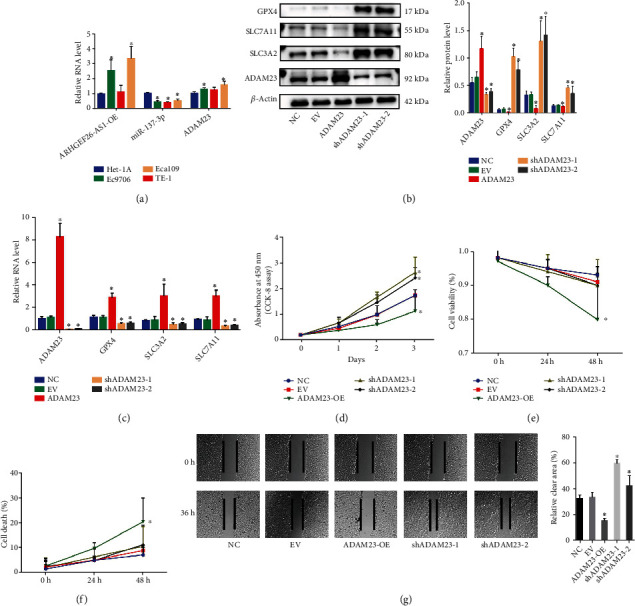
Overexpression of ADAM23 suppressed tumorigenesis and promoted ferroptosis in esophageal carcinoma cells. (a) Relative expression of ARHGEF26-AS1, miR-372-3p, and ADAM23 in ESCC cells; ^∗^*p* < 0.05; (b) Western blot results showing the expression of the ADAM23, GPX4, SLC7A11, and SLC3A2 proteins in TE-1 cells from the NC, EV, ADAM23-OE, shADMA23-1, and shADAM23-2 groups; ^∗^*p* < 0.05; (c) real-time PCR results showing the mRNA expression levels of ADAM23, GPX4, SLC3A2, and SLC7A11 in TE-1 cells following the overexpression and silencing of ADAM23. ^∗^*p* < 0.05; (d) the effect ADAM23 on cell proliferation, assessed through the CCK-8 assay. ^∗^*p* < 0.05; (e) cell viability and (f) cell death measured in subgroups with different levels of ADAM23 expression ^∗^*p* < 0.05; (g) proliferation and migration of TE-1 cells assessed through the wound healing assay. ^∗^*p* < 0.05.

ADAM23 and ARHGEF26-AS1 are tumour suppressor genes, and miR-372-3p may have a procarcinogenic effects (Supplementary Figure 2 B). However, there was no significant difference in the expression of ADAM23 in normal and esophageal cancer tissues; the expression of ARHGEF26-AS1 was higher in normal esophageal tissues than in esophageal cancer tissues (Supplementary Figure 3 A, B). The results in Figure 4(a) show that ARHGEF26-AS1 and ADAM23 were significantly highly expressed in the Ec9706, TE-1, and Eca109 cells compared to normal human oesophageal epithelial cells (Het-1A). Moreover, miR-137-3p was more highly expressed in the Het-1A and TE-1 cells. Therefore, TE-1 cells were used for the Western blot experiments and further analysis. In order to understand the functional significance of ADAM23 in ESCC cells, NC, EV, ADAM23, shADAM23-1, and shADAM23-2 groups were constructed. The Western blot experiments revealed that overexpression of ADAM23 downregulated the protein levels of the ferroptosis suppressor genes, GPX4, SLC7A11, and SLC3A2 (Figure 4(b)). On the other hand, silencing ADAM23 upregulated the protein levels of the ferroptosis suppressor genes, GPX4, SLC7A11, and SLC3A2 whose downregulation suggested the activation of the ferroptosis pathway. The RT-PCR results similarly suggested that overexpression of ADAM23 resulted in the upregulation of GPX4, SLC3A2, and SLC7A11 mRNA while silencing ADAM23 downregulated the mRNA expression of GPX4, SLC3A2, and SLC7A11 (*p* < 0.05, Figure 4(c) and Supplementary Figure 3C). These findings suggested that overexpression of ADAM23 promotes ferroptosis by downregulating the protein levels of GPX4, SLC7A11, and SLC3A2. However, overexpression of ADAM23 also promotes the mRNA expression of GPX4, SLC7A11, and SLC3A2. The results therefore suggested that ADAM23 may induce a feedback mechanism involving GPX4, SLC7A11, and SLC3A2, which inhibits ferroptosis and that this feedback mechanism may not reverse the ADAM23-induced depletion of GPX4, SLC7A11, and SLC3A2, ultimately leading to ferroptosis in ESCC cells. In order to test this hypothesis, CCK-8 assays were used to verify that overexpression of ADAM23 markedly inhibited the proliferation of ESCC cells while silencing ADAM23 significantly promoted the proliferation of ESCC cells (*p* < 0.05) (as shown in Figure 4(d) and Supplementary Figure 3D). Furthermore, cell viability and cell death were examined in different ADAM23 expression subgroups, at distinct periods (0, 24, and 48 h) (as shown in Figures 4(e) and 4(f) and Supplementary Figure 3E-F). The results revealed the presence of ESCC (TE-1) cells in the group where ADAM23 was overexpressed. Additionally, cell viability (%) was significantly downregulated while cell death (%) was markedly upregulated in the ADAM23-OE group. However, silencing ADAM23 significantly reversed this phenomenon. Moreover, wound healing assays were conducted to investigate the proliferation and migration of ESCC cells (Figure 4(g) and Supplementary Figure 3G). The findings showed that overexpression of ADAM23 (ADAM23-OE) significantly inhibited the proliferation and migration of ESCC cells. However, silencing ADAM23 (shADAM23-1/2) significantly promoted the proliferation and migration of ESCC cells. Overall, the results showed that overexpression of ADAM23 inhibited tumorigenesis and promoted ferroptosis in EC cells. Additionally, shADAM23-1 was found to have a stronger silencing capacity against ADAM23 and should therefore be explored further.

### 3.5. Upregulation of ADAM23 Enhanced Ferroptosis but Restrained Cell Growth in miR-372-3p-Regulated ECC

**Figure 5:**
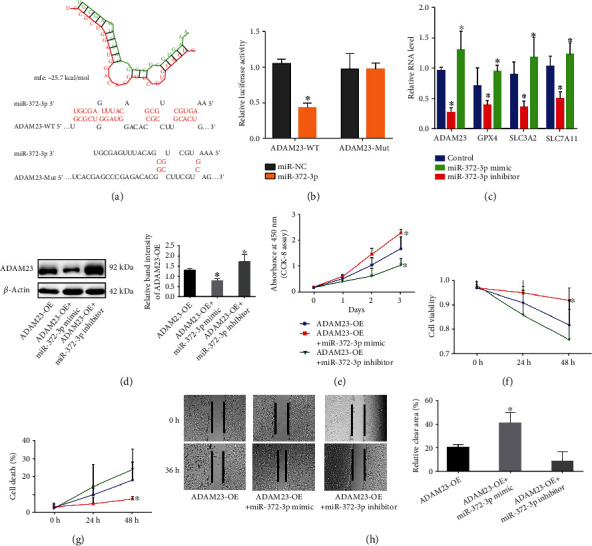
Upregulation of ADAM23 enhanced ferroptosis and inhibited cell growth in esophageal carcinoma cells through the downregulation of miR-372-3p. (a) The binding of miR-372-3p on ADAM23 as predicted by RNAhybrid 2.12. (b) Regulation of ADAM23 by miR-372-3p, assessed through luciferase reporter assays. ^∗^*p* < 0.05 vs. miR-NC. (c) The effect of up- and downregulation of miR-372-3p on the expression level of ADAM23, assessed through the WB assay; ^∗^*p* < 0.05; (d) real-time PCR results showing the mRNA expression levels of GPX4, SLC3A2, and SLC7A11 in TE-1 cells cotransfected with the miR-372-3p mimic or miR-372-3p inhibitor after the overexpression of ADAM23; ^∗^*p* < 0.05; (e) the effect of the miR-372-3p mimic and miR-372-3p inhibitor on cell proliferation after the overexpression of ADAM23, assessed through the CCK-8 assay. ^∗^*p* < 0.05; (g) the effect of the miR-372-3p mimic and miR-372-3p inhibitor on (f) cell proliferation and (g) cell death after the overexpression of ADAM23; ^∗^*p* < 0.05; (h) the proliferation and migration of TE-1 cells assessed through the wound healing assay. ^∗^*p* < 0.05.

The effect of ADAM23 on EC and its correlation with miR-372-3p have not yet been investigated. In this study, the results from Bioinformatics analysis and the luciferase reporter gene assay suggested that miR-372-3p could interact with ADAM23 (Figures 5(a) and 5(b)). In addition, the Western blot experiments confirmed that the upregulation of miR-372-3p could downregulate the protein levels of ADAM23 (Figure 5(c)). Moreover, the RT-PCR results revealed that upregulation of miR-372-3p resulted in a decrease in the mRNA levels of ADAM23, GPX4, SLC3A2, and SLC7A11 (Figure 5(d) and Supplementary Figure 3C). The findings therefore suggested that miR-372-3p is an upstream regulator of ADAM23. Additionally, the CCK-8 assay showed that cell viability was significantly upregulated in the miR-372-3p mimic group compared to that in the miR-372-3p inhibitor category and ADAM23 overexpression control group (Figure 5(e) and Supplementary Figure 3D). Further analyses also showed that cell viability (%) was significantly upregulated while cell death (%) was markedly downregulated in the miR-372-3p mimic group. However, this was reversed in the miR-372-3p inhibitor group (Figures 5(f) and 5(g) and Supplementary Figure 3E, F). Furthermore, the proliferation and migration of TE-1 cells were assessed through the wound healing assay (Figure 5(h) and Supplementary Figure 3G). While there was an in increase in the proliferation and migration of ESCC cells in the miR-372-3p mimic group, proliferation and migration were slowed down in the miR-372-3p inhibitor category. Overall, these results suggested that upregulation of ADAM23 enhanced ferroptosis but the restrained cell growth in ECC was caused by miR-372-3p.

### 3.6. LncRNA ARHGEF26-AS1 Facilitated Ferroptosis but Restrained Cell Growth and Positively Regulated ADAM23 by Sponging miR-372-3p in ECC

**Figure 6:**
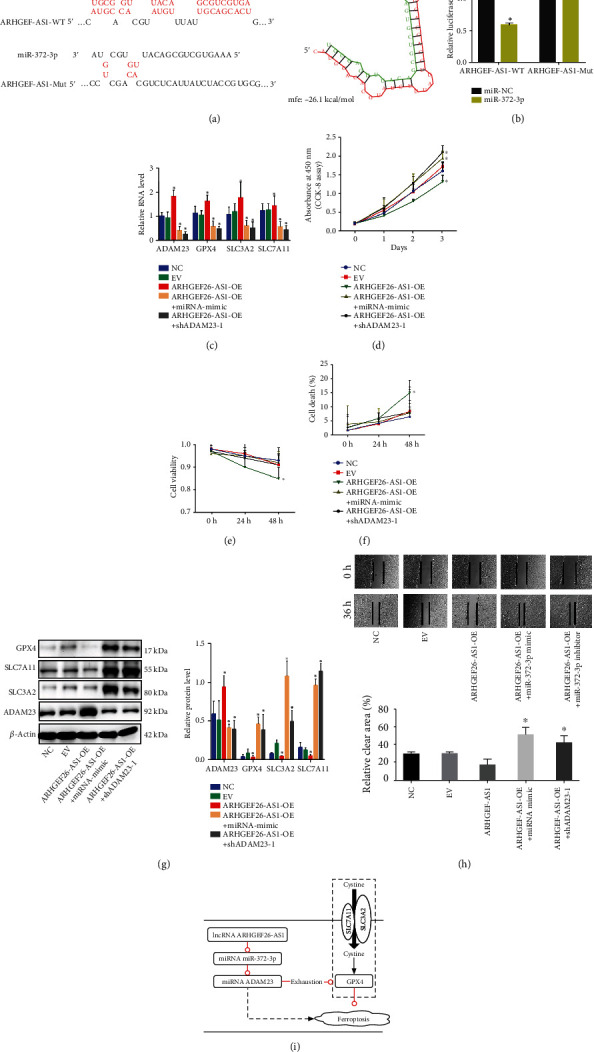
The ARHGEF26-AS1 lncRNA promoted ferroptosis, inhibited cell growth and upregulated the expression of ADAM23 by sponging miR-372-3p in esophageal carcinoma cells. (a) The binding of miR-372-3p to ARHGEF26-AS1, predicted with RNAhybrid 2.12. (b) Regulation of miR-372-3p by ARHGEF26-AS1, assessed through luciferase reporter assays. ^∗^*p* < 0.05 vs. miR-NC.; (c) the relative mRNA levels of ADAM23, GPX4, SLC3A2, and SLC7A11 in TE-1 cells cotransfected with the miR-372-3p mimic or shADAM23-1, following the overexpression of ARHGEF26-AS1. Data is presented as the mean ± SD. ^∗^*p* < 0.05; (d) the effect of the miR-372-3p mimic and shADAM23-1 on cell proliferation after the overexpression of ARHGEF26-AS1, assessed through the CCK-8 assay. ^∗^*p* < 0.05; (e, f) the effect of ARHGEF26-AS1 overexpression on (e) cell proliferation and (f) cell death after treatment with either the miR-372-3p mimic or shADAM23-1. ^∗^*p* < 0.05; (g) verification of ARHGEF26-AS1 overexpression through Western blotting following the overexpression of miR-372-3p and the effect of shADAM23-1 on the expression of ADAM23; ^∗^*p* < 0.05; (h) proliferation and migration of TE-1 cells, assessed through the wound healing assay; (i) effect of the ARHGEF26-AS1/miR-372-3p/ADAM23 pathway on ferroptosis. ^∗^*p* < 0.05.

**Figure 7:**
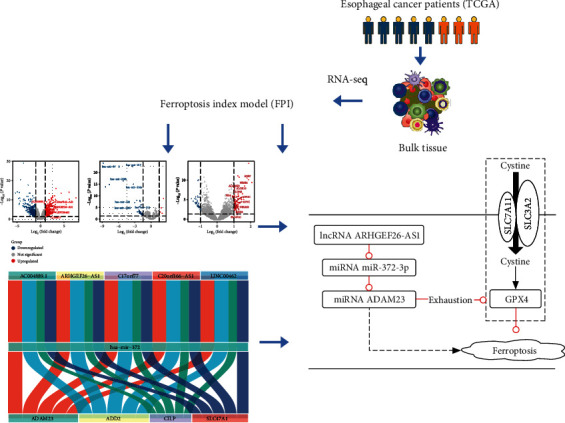
Graphical abstract image.

Bioinformatics analysis suggested potential binding between miR-372-3p and ARHGEF26-AS1 (Figure 6(a)). Consequently, the regulation of miR-372-3p by ARHGEF26-AS1 was assessed using luciferase reporter assays (Figure 6(b)). The findings revealed that overexpression of ARHGFE26-AS1 was associated with an increase in the mRNA levels of ADAM23, GPX4, SLC3A2, and SLC7A11 (Figure 6(c)). As expected, the cotransfected miR-372-3p mimic or shADAM23-1 after the overexpression of ARHGEF26-AS1 led to the downregulation of ADAM23, GPX4, SLC3A2, and SLC7A11. Moreover, the CCK-8 assay was used to examine the effects of the miR-372-3p mimic and shADAM23-1 on cell proliferation after the overexpression of ARHGEF26-AS1 (Figure 6(d)). The effects of the miR-372-3p mimic and shADAM23-1 on cell viability and cell death were also examined following the overexpression of ARHGEF26-AS1 (Figures 6(e) and 6(f)). Similar to the overexpression of ADAM23, there was an increase in cell proliferation, migration, and viability while cell death was inhibited following the overexpression of ARHGEF26-AS1. Moreover, there was a decrease in the proliferation, migration, and viability of TE-1 cells in the miR-372-3p mimic and shADAM23-1 groups, after the overexpression of ARHGEF26-AS1. There was also an increase in cell death. The study further validated the potential role of ARHGEF26-AS1 overexpression in the ferroptosis pathway, at the protein level (Figure 6(g)). Therefore, the wound healing assay was used to examine the proliferation and migration of TE-1 cells (Figure 6(h)). The results showed that overexpression of ARHGEF26-AS1 upregulated the protein levels of ADAM23 but depleted the protein levels of GPX4, SLC3A2, and SLC7A11. In summary, the findings revealed that the lncRNA, ARHGEF26-AS1 facilitated ferroptosis but restrained cell growth and positively regulated ADAM23 by sponging miR-372-3p in ECC (Figures 6(i) and 7).

## 4. Discussion

The present study for the first time identified and constructed a potential ferroptosis-related ceRNA network based on FPI scores. In addition, the ARHGEF26-AS1/miR-372-3p/ADAM23 pathway promoted the ferroptosis pathway and inhibited the growth of ESCC cells. The study also identified a potential mechanism in which the overexpression of ADAM23 could deplete the protein levels of GPX4, SLC3A2, and SLC7A11, hence promoting ferroptosis.

This study revealed that overexpression of ADAM23 not only promoted cell death but also reduced the proliferation, migration, and viability of cells. The ADAM (a disintegrin and metalloprotease) family includes both protein-hydrolyzing and nonprotein-hydrolyzing members, which have been associated with numerous biological events, such as membrane protein shedding, migration, cell adhesion, and protein hydrolysis [19]. Notably, ADAM23 is a member of the nonproteolytic ADAM family of genes. It was previously reported that low expression or hypermethylation of ADAM23 was associated with poor prognosis in lung, ovarian, breast, and gastric cancers [19–22]. In addition, ADAM22 was shown to be associated with tolerance to chemotherapy, in breast cancer [23]. It is also noteworthy that ADAM22 is highly homologous to ADAM23, and the two are thought to have similar tumour suppressor functions. In the current study, overexpression of ADAM23 resulted in the depletion of GPX4, SLC3A2, and SLC7A11, at the protein level. Interestingly, overexpression of ADAM23 upregulated the mRNA levels of GPX4, SLC3A2, and SLC7A11. This suggests that ADAM23 may induce a feedback mechanism involving GPX4, SLC7A11, and SLC3A2, which inhibits ferroptosis and that this feedback mechanism may not reverse the ADAM23-induced depletion of GPX4, SLC7A11, and SLC3A2, ultimately leading to ferroptosis in ESCC cells. It is therefore possible that the depletion of GPX4, SLC3A2, and SLC7A11, induced by the overexpression of ADAM23, is a potential mechanism for the development of ferroptosis [24]. Moreover, regulation of cystine uptake by SLC7A11 and SLC3A2 is a key step in the biosynthesis of glutathione [25, 26]. On the other hand, GPX4 is a key cellular antioxidant enzyme, and its depletion or dysfunction leads to the intracellular accumulation of reactive oxygen species (ROS) and lipid peroxidation [27]. Herein, the results suggested that overexpression of ADAM23 might induce ferroptosis through the depletion of GPX4, SLC3A2, and SLC7A11.

The present study identified ferroptosis-associated DE-lncRNAs, DE-miRNAs, and DE-mRNAs in ESCC, then constructed a survival-related ceRNA network using clinical data from the TCGA database. Notably, the lncRNA ARHGEF26-AS1/miR-372-3p/ADAM23 pathway was found to be the most promising ferroptosis-related pathway, based on correlation and survival analyses. Over the recent years, studies have successively demonstrated that lncRNAs can regulate tumour ferroptosis [28, 29]. However, no study has shown that lncRNA and miRNA regulate ferroptosis in EC. Moreover, previous studies showed that miR-372 was highly expressed in EC tissues [30]. In the present study, the results revealed that high expression of miR-372 was associated with poor survival of EC patients. Furthermore, miR-372-3p was reported to promote tumour growth in rectal cancer [31]. Similarly, miR-372-3p was shown to promote tumour growth and metastasis in lung squamous cell carcinoma [32]. miRNA-372-3p was also reported to promote epithelial-mesenchymal transition in breast cancer by upregulating the Wnt pathway [33]. The current study for the first time uncovered that miR-372-3p has a prooncogenic role in ESCC as it promotes the proliferation and migration of ESCC cells.

Furthermore, this is the first study to reveal that lncRNA regulates ESCC through a ferroptosis-related mechanism. The study also showed that low expression of ARHGEF26-AS1 was associated with the poor prognosis of EC. Although several studies have revealed that ARHGEF26-AS1 may be involved in multiple processes in cancer through the ceRNA network, the mechanism through which ARHGEF26-AS1 regulates the development of cancer has not been experimentally confirmed [34–36]. Herein, the luciferase reporter assays confirmed that ARHGEF26-AS1 can upregulate the expression of ADAM23 by competitively binding to miR-372-3p. Similar to the overexpression of ADAM23, overexpression of ARHGEF26-AS1 promoted cell proliferation, migration, and viability but inhibited cell death. Further cellular experiments also revealed that overexpression of ARHGEF26-AS1 promoted ferroptosis by upregulating the protein levels of ADAM23 and lowering the protein levels of GPX4, SLC3A2, and SLC7A11.

Our study hints at the positive implications of targeted therapy with ADAM23 for the treatment of EC. In the current study, ADAM23 was shown to be a receptor for the neuropeptide LGI1 [37]. LGI1, a tumour suppressor, inhibits the growth and metastasis of breast cancer by target binding to ADAM23/AMAM22 [23]. A number of neuropeptides have been reported to be associated with the development of ferroptosis [38]. And neuropeptides are key mediators in the exacerbation or onset of depression [39]. Depression has been found to be a significant cause of poor prognosis in patients with EC [40]. In addition, depression potentially leads to downregulated LGI1 [41]. We hypothesized that, in the microenvironment of oesophageal cancer tissues, a depressive state would upregulate ADAM23 by downregulating LGI1 expression. In conclusion, a neuropeptide-related ferroptosis regulatory pathway was identified in this study.

This study therefore revealed that ARHGEF26-AS1 is the mechanism through which miR-372-3p regulates the expression of ADAM23, thus inhibiting the proliferation and migration of ESCC cells and upregulating the ferroptosis pathway. Due to experimental constraints, only markers of ferroptosis and one cell line TE-1 were detected, but no cellular phenotype related to ferroptosis was verified. However, more studies (including more cell line studies and phenotype validation) should be conducted to uncover the mechanisms through which ferroptosis regulates carcinogenesis and development of oesophageal cancer. Moreover, the effect of the selected genes in this ceRNA regulatory network on the ferroptosis pathway in EC needs to be validated through more experiments. Animal studies and other *in vitro* experiments related to the ARHGEF26-AS1/miR-372-3p/ADAM23 pathway should also be conducted. In addition, clinical studies are needed to elucidate the mechanism through which ADAM23 regulates ferroptosis in EC.

## 5. Conclusions

The findings revealed that the lncRNA, ARHGEF26-AS1 acts as a miR-372-3p sponge that regulates the expression of ADAM23, thus inhibiting the proliferation of ESCC cells and upregulating the ferroptosis pathway. This may therefore reveal a new set of therapeutic targets for EC.

## Data Availability

Contact the corresponding author to provide all raw data.

## Abbreviations

ceRNA: Competing endogenous RNAs
DE-mRNAs: Differentially expressed mRNAs
DE-miRNAs: Differentially expressed micro-RNAs
DE-lncRNAs: Differentially expressed lncRNAs
EV: Empty vector
ESCC: Oesophageal squamous cell carcinoma
FPI: Ferroptosis Potential Index
GO: Gene Ontology
GSEA: Gene Set Enrichment Analysis
KEGG: Kyoto Encyclopedia of Genes and Genomes
K-M: Kruskal-Wallis
OS: Overall survival
RT-PCR: Quantitative real-time PCR
TCGA: The Cancer Genome Atlas.

## Additional Points

*Key points*. Significant findings from the study: the Ferroptosis Potential Index (FPI), reflecting the activity of the ferroptosis pathway, was determined, and a ferroptosis-associated ceRNA network was established. The findings revealed that the lncRNA, ARHGEF26-AS1 acts as a miR-372-3p sponge that regulates the expression of ADAM23. Significance of the study: this is the first study to reveal that lncRNA plays a role in esophageal squamous cell carcinoma (ESCC) through a mechanism related to ferroptosis. The findings showed that ARHGEF26-AS1 acts as a miR-372-3p sponge that regulates the expression of ADAM23, inhibiting the proliferation of ESCC cells and upregulating the ferroptosis pathway.

## Supplementary Materials

Supplementary Materials All supplementary material was included in the file “Supplementary methods and figures.doc” which describes the supplementary methods and supplementary Figures 1 to 3.
