# Supplementary material for: Mechanism and Role of the Neuropeptide LGI1 Receptor ADAM23 in Regulating Biomarkers of Ferroptosis and Progression of Esophageal Cancer
Source: Dis Markers. 2021 Dec 30;2021:9227897. doi: 10.1155/2021/9227897 (PMC8739919; doi:10.1155/2021/9227897)
Supplement: Supplementary Materials — All supplementary material was included in the file “Supplementary methods and figures.doc” which describes the supplementary methods and supplementary Figures 1 to 3. [file 9227897.f1.doc]

## SUPPLEMENTARY METHODS AND FIGURES

**SUPPLEMENTARY METHODS**

**Overexpression and knockdown of ADAM23**

ADAM23 expression vectors and empty vectors were purchased from Origene (Rockville, MD, USA) and transfected using FuGENE6 (Roche Diagnostics). For knockdown of ADAM23, the two different lentiviral vectors for ADAM23 shRNA and non-targeting shRNA vector were purchased from Sigma-Aldrich. The coding sequences of the shRNA were as follows: sh1, 5’-CCGGGCAGTGTTACTGGAACTATTACTCGAGTAATAGTTCCAGTAACACTGCTTTTTG-3’; sh2, 5-CCGGCCACTCGATTCCAAGGGTAAACTCGAGTTTA- -CCCTTGGAATCGAGTGGTTTTTG-3’. The stable transfectants were selected as described previously ^1,2^.

**Quantitative real-time RT-PCR**

Total RNA was isolated from samples using the RNeasy Plus Mini kit (Qiagen) then reverse transcribed into cDNA using the superscript first-strand synthesis SuperMix kit (Invitrogen). RT-PCR analysis was performed using SYBR Green. The following primers were used: ADAM23, forward 5’-TACAATGG- -CGAGTGCAAGAC-3’, reverse 5’-GCAGTTTCCCTTCTCAGTGC-3’; GPX4, forward 5’-ACAAGAACGGCTGCGTGGTGAA-3’, reverse 5’-GCCACACA- -CTTGTGGAGCTAGA-3’; SLC3A2, forward 5’-CCAAGGTGAAGGATGC- -TCTG-3’, reverse 5’-TGTGTGACTAGGGATTTTGTATGC-3’; SLC7A11, forward 5’-ATGCAGTGGCAGTGACCTTT-3’, reverse 5’-GGCAACAAAGATCGGAAC- -TG-3’.

**Wound Healing assay**

Cells were grown to the appropriate confluence in 6-well plates. The wound was created with a sterile plastic tip. After washing with PBS, cells were cultured for 24 h in serum-free medium. Images were taken using a microscope. An average of six random widths of each wound was measured for quantitation.^3^

#
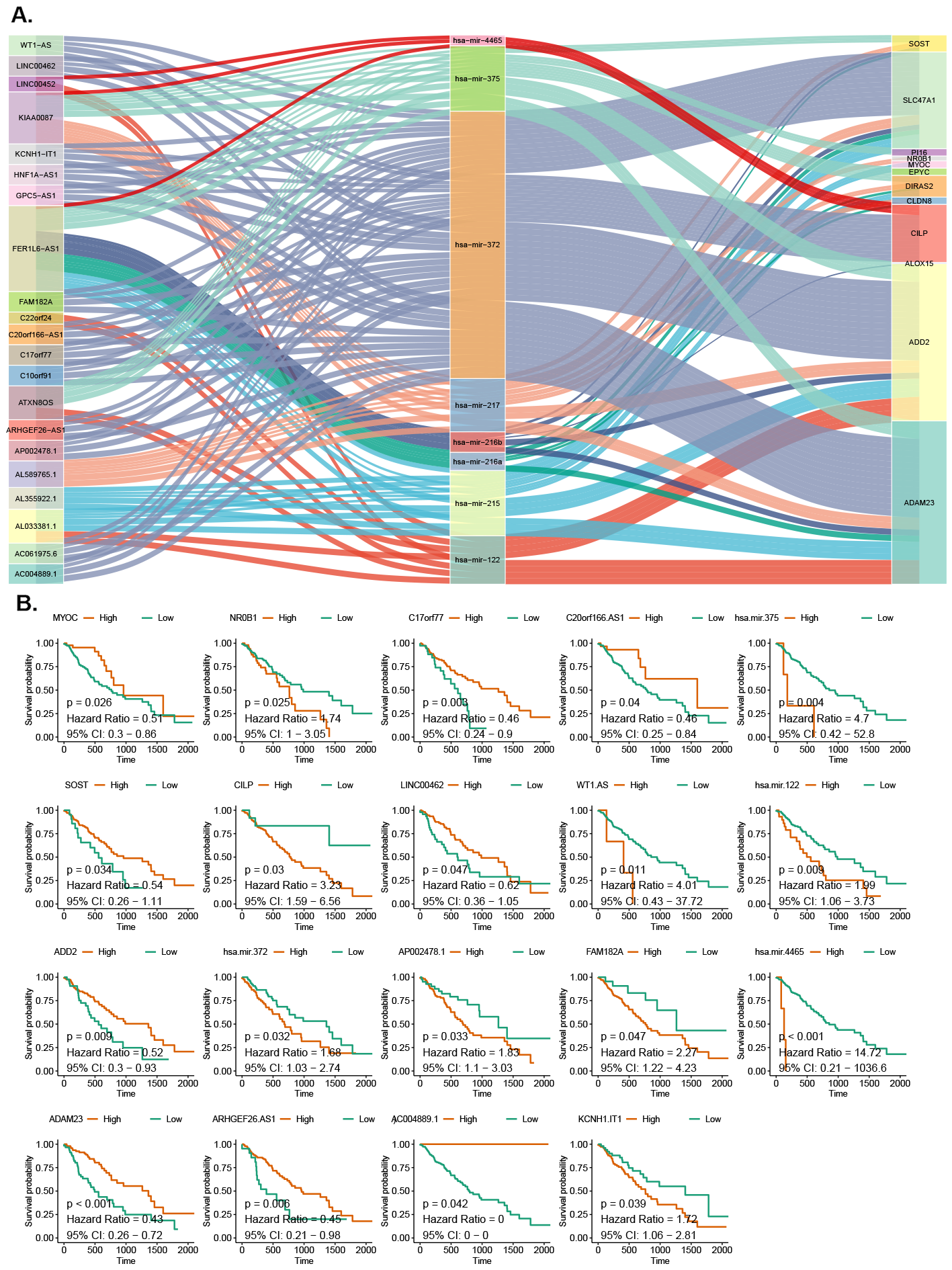
SUPPLEMENTARY FIGURES

**Supplementary Figure 1. The Established ceRNA network and Survival Analysis**

1. A ceRNA network of esophageal cancer and ferroptosis;
2. K-M survival analysis of OS based on DE-lncRNAs, DE-miRNAs and DE-mRNAs in the TCGA cohort.


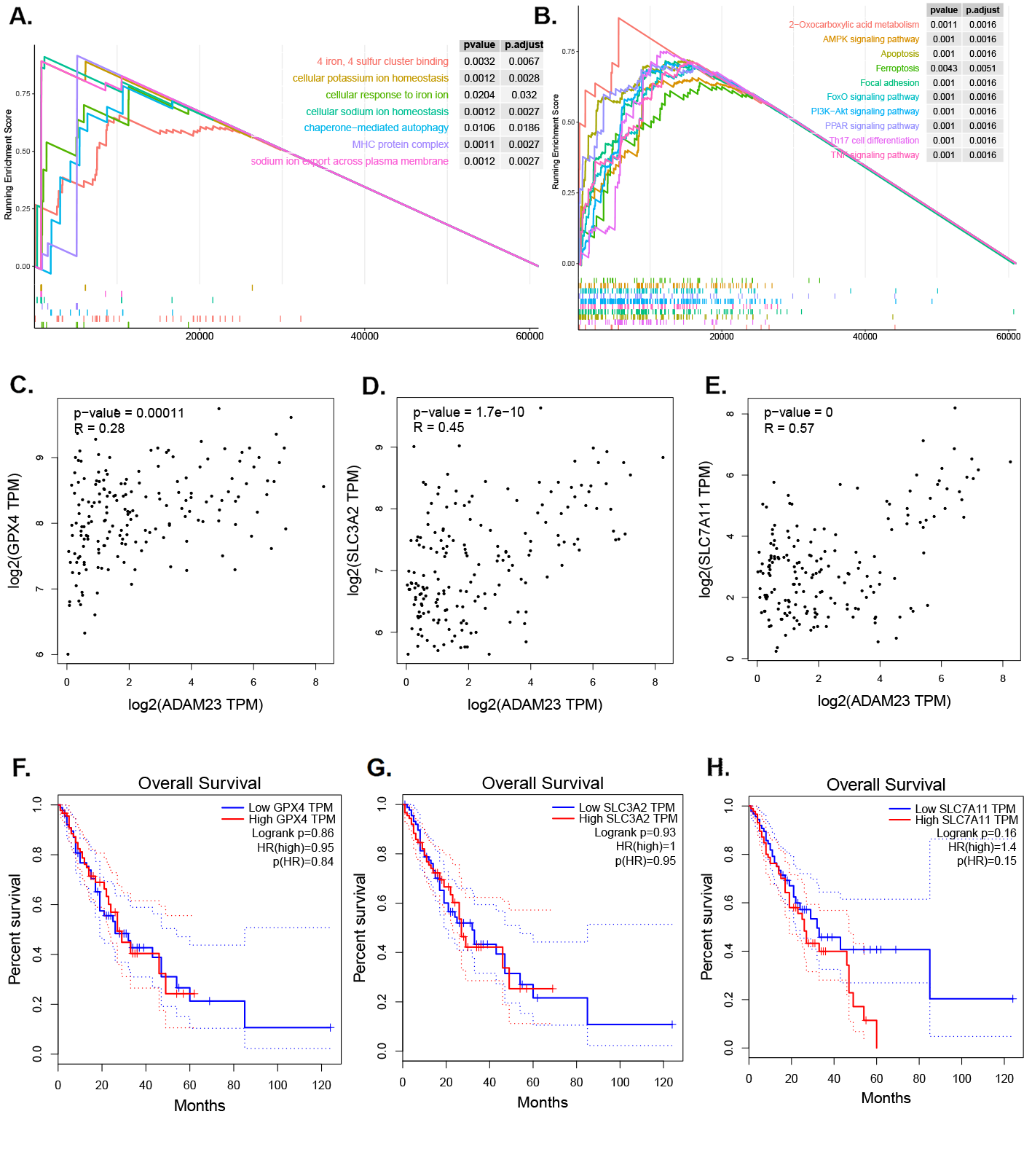


**Supplementary Figure 2.**

A-B. GSEA showing (A) GO function analysis and (B) KEGG pathway analysis
of ADAM23.

C-E. Correlation between the ferroptosis-related genes (C) GPX4 (D) SLC3A2 (E) SLC7A11 and ADAM23, respectively.

F-G. Survival analysis of the ferroptosis-related genes (GPX4, SLC3A2 and SLC7A11).


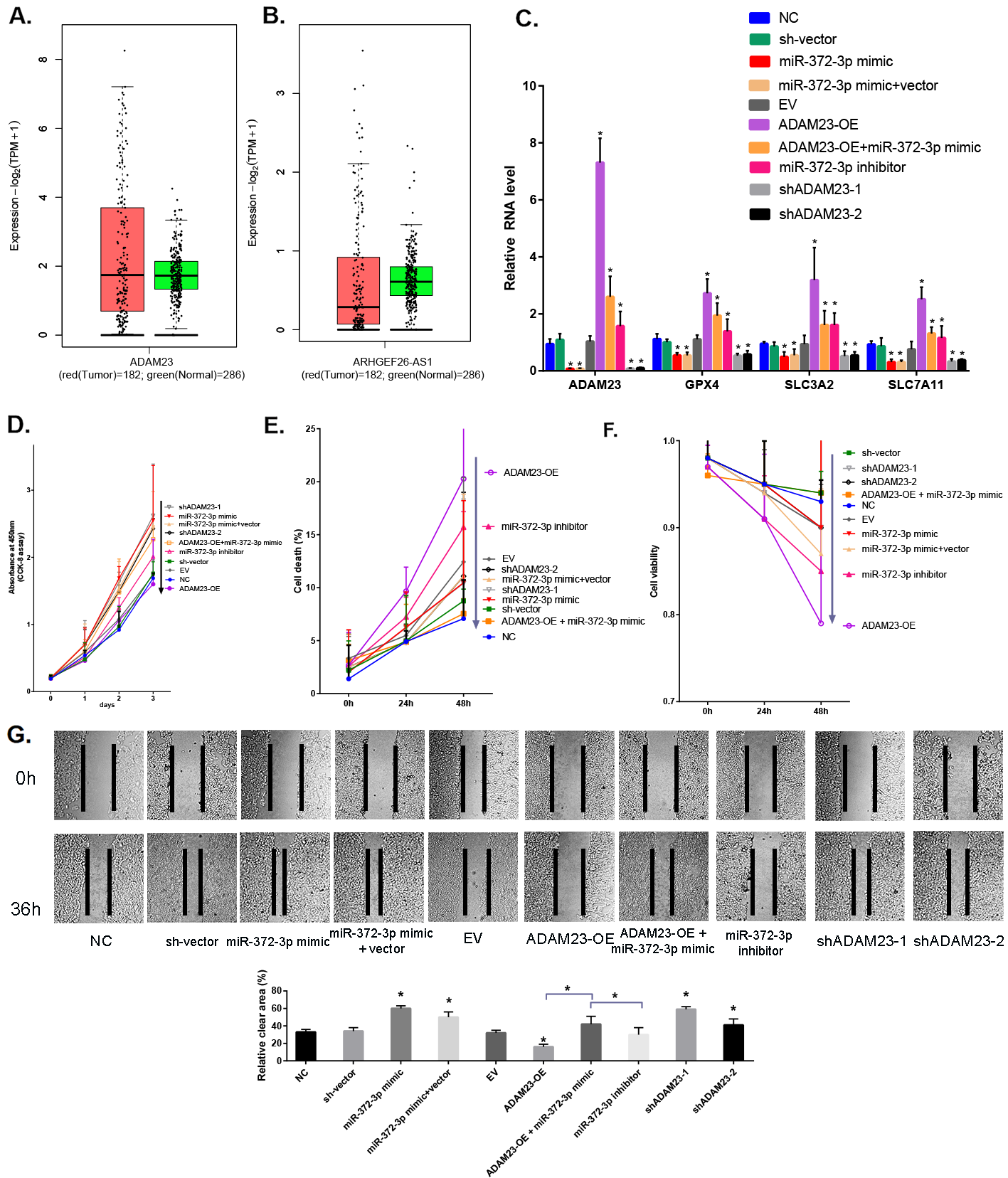


**Supplementary Figure 3.**

A-B. Exploring the expression of ADAM23 and ARHGEF26-AS1 in normal and esophageal cancer patients based on GEPIA.

C. Real-time PCR results showing the mRNA expression levels of ADAM23, GPX4, SLC3A2 and SLC7A11 in TE-1 cells. * p < 0.05;

D. The effect ADAM23 on cell proliferation, assessed through the CCK-8 assay. ***** p < 0.05;

E-F. (E) Cell viability and (F) cell death measured in subgroups with different levels of ADAM23 expression ***** p < 0.05;

G. Proliferation and migration of TE-1 cells assessed through the wound healing assay. ***** p < 0.05.

**References**

1. Ota M, Mochizuki S, Shimoda M, Abe H, Miyamae Y, Ishii K, Kimura H, Okada Y. ADAM23 is downregulated in side population and suppresses lung metastasis of lung carcinoma cells. Cancer Sci. 2016 Apr;107(4):433-43.

2. Mochizuki S, Soejima K, Shimoda M, Abe H, Sasaki A, Okano HJ, Okano H, Okada Y. Effect of ADAM28 on carcinoma cell metastasis by cleavage of von Willebrand factor. J Natl Cancer Inst. 2012 Jun 20;104(12):906-22.

3. Hou H, Zhao H, Yu X, Cong P, Zhou Y, Jiang Y, Cheng Y. METTL3 promotes the proliferation and invasion of esophageal cancer cells partly through AKT signaling pathway. Pathol Res Pract. 2020 Sep;216(9):153087.
